# Post-dural Puncture Headache During Bacteraemia: Successful Management With Combined Sphenopalatine Ganglion and Greater Occipital Nerve Blocks

**DOI:** 10.7759/cureus.111795

**Published:** 2026-06-30

**Authors:** Leonardo Monteiro, João V Pais, Anabela S Vieira, Mariana Flor de Lima, Sara Mota

**Affiliations:** 1 Anaesthesiology, Unidade Local de Saúde do Tâmega e Sousa, Penafiel, PRT

**Keywords:** bacteraemia, epidural blood patch, greater occipital nerve block, post-dural puncture headache, sphenopalatine ganglion block

## Abstract

Epidural blood patch (EBP) is considered the most effective interventional treatment for post-dural puncture headache (PDPH), but it is considered a relative contraindication during active bacteraemia because of the theoretical risk of neuraxial seeding. We describe a 26-year-old primiparous woman who developed a typical orthostatic headache after a technically difficult labour epidural analgesia. On postpartum day 4, she developed fever and urinary symptoms; blood and urine cultures grew *Escherichia coli,* and abdominal imaging subsequently confirmed acute right pyelonephritis with bacteraemia. Brain magnetic resonance imaging (MRI) with venographic sequences excluded cerebral venous sinus thrombosis and other intracranial pathology. After multidisciplinary discussion, we performed bilateral transnasal sphenopalatine ganglion block followed by bilateral ultrasound-guided greater occipital nerve block on postpartum day 6. Headache severity decreased from a numerical rating scale (NRS) of 7/10 to 2/10 within 30 minutes, and the patient was able to resume care of her newborn. She remained symptom-free at hospital discharge and at telephone follow-up 7 and 30 days later. This case supports peripheral nerve blocks as a minimally invasive bridge therapy for PDPH when EBP is contraindicated, deferred or declined, and adds to the limited literature on documented obstetric bacteraemia.

## Introduction

Post-dural puncture headache (PDPH) is an important complication of neuraxial procedures and, in obstetrics, of labour epidural analgesia. Accidental dural puncture complicates approximately 1% of labour epidural procedures, and around half of affected patients develop PDPH [1,2]. Conservative measures (bed rest, oral hydration, caffeine and simple analgesia) are usually attempted first [3], but EBP is recommended by the 2023 Multisociety International Consensus Guidelines as the most effective interventional treatment for moderate-to-severe symptoms or symptoms refractory to conservative therapy [4]. The same guidelines identify active systemic infection, including bacteraemia, as a relative contraindication to EBP because of the theoretical risk of meningeal or epidural seeding when autologous blood is injected into the neuraxis [4]. Although direct evidence of harm is limited to isolated case reports [5], this contraindication creates a difficult clinical scenario: the headache may be disabling, yet the most effective interventional treatment must be deferred while systemic infection is treated and alternative intracranial pathology is excluded [4].

Sphenopalatine ganglion block (SPGB), with or without greater occipital nerve block (GONB), has been described as a minimally invasive treatment modality for PDPH in obstetric and other settings [4,6-9]. The proposed mechanisms - modulation of parasympathetic outflow from the pterygopalatine (sphenopalatine) ganglion and reduction of trigeminocervical nociceptive input - differ from the dural sealing achieved by EBP, and these blocks should not be regarded as an equivalent alternative to EBP. However, they may offer useful symptom control when neuraxial intervention is contraindicated, deferred or declined [4,6-9]. We describe the use of bilateral transnasal SPGB followed by bilateral ultrasound-guided GONB in a postpartum patient with typical PDPH and documented *Escherichia coli* bacteraemia.

## Case presentation

A 26-year-old primiparous woman (height 169 cm, weight 88 kg, body mass index (BMI) 30.8 kg/m²; American Society of Anesthesiologists (ASA) physical status 2) was admitted at 39+6 weeks of gestation after spontaneous rupture of membranes. Her antenatal course was notable only for the treated asymptomatic bacteriuria in the second trimester, with subsequent negative urine culture. Past medical history was otherwise unremarkable.

Labour epidural analgesia was attempted at the L3-L4 interspace with an 18-G Tuohy needle (Portex; Smiths Medical, Hythe, Kent, UK) using a midline approach. Identification of the epidural space was technically difficult and required three attempts. No cerebrospinal fluid (CSF) or blood was aspirated from the Tuohy needle or, subsequently, from the epidural catheter, and no paraesthesiae were reported. Correct epidural placement was confirmed by loss of resistance to saline, with a satisfactory bilateral sensory block to the T10 dermatome. A fractionated bolus of 10 mL of 0.2% ropivacaine with 10 μg of sufentanil produced satisfactory analgesia. Vacuum-assisted vaginal delivery was performed due to maternal exhaustion, and a healthy neonate was delivered.

On postpartum day 2, the patient developed a bilateral frontal, postural headache with mild photophobia that began within minutes of standing and resolved when supine. Headache severity in the upright position was a numerical rating scale (NRS) score of 5/10. The clinical picture fulfilled International Classification of Headache Disorders, third edition (ICHD-3) criteria for headache attributed to low CSF pressure [10]. She was apyrexial and haemodynamically stable. Conservative treatment with bed rest, oral hydration (encouraged to approximately 1.5 L/day), oral caffeine 300 mg daily, intravenous (IV) paracetamol 1 g six-hourly and ketorolac 30 mg six-hourly, with IV tramadol 100 mg eight-hourly as required, produced only transient improvement. These measures were continued from postpartum day 2 to postpartum day 4.

On postpartum day 4, she developed fever (peak temperature 38.4°C) and urinary symptoms. She remained haemodynamically stable, with a blood pressure of 118/62 mmHg, heart rate of 87 beats/min, respiratory rate of 13 breaths/min and peripheral oxygen saturation of 97% on room air. Laboratory testing showed leucocytosis with neutrophilia and a markedly raised C-reactive protein (CRP) concentration (Table 1). Procalcitonin and presepsin were not measured. Urinalysis showed pyuria and nitrites, and blood and urine cultures subsequently grew *E. coli,* confirming acute right pyelonephritis with bacteraemia; the diagnosis was later corroborated by abdominal computed tomography (CT) on postpartum day 7. Intravenous ceftriaxone (1 g) once daily was started and continued in accordance with susceptibility testing.

**Table 1:** Laboratory findings on postpartum day 4 Reference ranges are those of the reporting hospital laboratory. The absolute neutrophil count corresponds to 93% of the total leukocyte count.

Parameter	Result	Reference range
Leukocytes	23.7 × 10⁹/L	4.5-11.0 × 10⁹/L
Neutrophils	22.0 × 10⁹/L (93%)	2.0-7.5 × 10⁹/L
C-reactive protein	150.6 mg/L	< 5.0 mg/L

By postpartum day 6, the headache remained incapacitating (NRS 7/10) and limited maternal functioning. Brain magnetic resonance imaging (MRI) with venographic sequences was performed to exclude cerebral venous sinus thrombosis and other postpartum intracranial pathology. The study was reported as normal: there were no intracranial haemorrhagic or recent ischaemic lesions, no pericerebral collections and no expansive lesions. Parenchymal morphology and signal were normal, the CSF spaces were of normal morphology and dimensions, and the cerebellar tonsils were normally positioned and the basal cisterns patent. The venous sinuses showed a normal flow void without evidence of thrombosis, and the paranasal sinuses and middle ears were aerated. No diagnostic image was available for inclusion as a figure. 

Lumbar puncture for CSF analysis was not performed, in view of the active bloodstream infection, the typical orthostatic pattern of the headache and the absence of clinical signs of meningism. 

The differential diagnosis of postpartum headache is broad, and primary and secondary causes were considered systematically [11]. Pre-eclampsia and eclampsia were considered unlikely given consistently normal blood pressure and the absence of other suggestive features. Cerebral venous sinus thrombosis, intracranial haemorrhage and other acute intracranial pathology - including posterior reversible encephalopathy syndrome and reversible cerebral vasoconstriction syndrome - were excluded by the normal MRI with venographic sequences. Bacterial meningitis, although relevant in the context of bacteraemia, was considered unlikely given the strictly orthostatic, posture-dependent characteristic of the headache, the absence of meningism and a pattern that fluctuated with posture rather than with fever. Primary headache disorders (migraine and tension-type headache) and a sinonasal (ear, nose and throat) cause were considered unlikely in the absence of any relevant previous headache history and given the aerated paranasal sinuses and middle ears on imaging. The strongly orthostatic phenotype, the temporal relationship to technically difficult neuraxial instrumentation, fulfilment of ICHD-3 criteria [10] and the rapid, sustained response to targeted blocks together supported a clinical diagnosis of PDPH.

Because the clinical picture remained highly suggestive of PDPH while documented bacteraemia made EBP undesirable, we proceeded - after multidisciplinary discussion involving obstetric anaesthesia, obstetrics and infectious diseases - with bilateral transnasal SPGB followed by bilateral ultrasound-guided GONB. The rationale, alternatives, expected effects and potential adverse effects of the blocks were explained to the patient, and verbal consent was obtained separately from the consent for publication.

**Figure 1:**
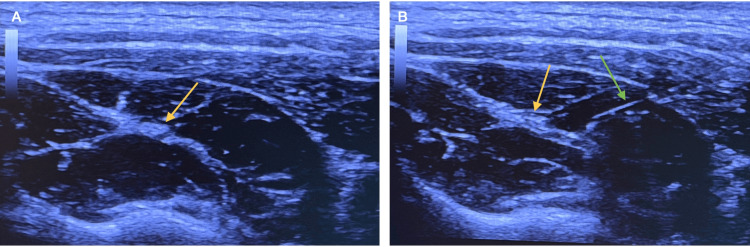
Ultrasound-guided greater occipital nerve block at the level of the obliquus capitis inferior muscle. (A) Sonographic identification of the greater occipital nerve (yellow arrow) before needle insertion. (B) In-plane advancement of the block needle (green arrow) towards the greater occipital nerve (yellow arrow).

The blocks were performed at the bedside with standard non-invasive monitoring (electrocardiography, non-invasive blood pressure and pulse oximetry). Ropivacaine was used for both blocks; the concentrations and volumes were chosen in line with published descriptions of sphenopalatine ganglion and greater occipital nerve blocks for PDPH, in which a range of local anaesthetics (including lignocaine and ropivacaine) and volumes of approximately 1-3 mL per side have been reported [7,9,12]. The transnasal SPGB was performed using a cotton-tipped applicator soaked in 2 mL of 0.75% ropivacaine introduced into each nostril, directed posteriorly along the floor of the nasal cavity towards the posterior nasopharyngeal wall, aiming to reach the mucosa overlying the sphenopalatine ganglion in the pterygopalatine fossa. The applicators were left in place for 5 minutes per side. The greater occipital nerves were then identified bilaterally between the semispinalis capitis and obliquus capitis inferior muscles using a high-frequency linear ultrasound transducer (3-12 MHz) and blocked with 3 mL of 0.375% ropivacaine per side (Figure 1). The blocks were well tolerated, with no epistaxis, lacrimation, local discomfort or other adverse effects.

Headache severity decreased from NRS 7/10 to NRS 2/10 within 30 minutes of the blocks, and the patient was able to sit and stand without recurrence. On postpartum day 7, she developed recurrent fever; abdominal CT confirmed acute right pyelonephritis. Repeat blood cultures became negative and ceftriaxone was continued. No further intervention for headache was required, and EBP was not subsequently offered as the patient remained asymptomatic and clinically improving. She was discharged on postpartum day 15 and was symptom-free at telephone follow-up 7 and 30 days after discharge. The clinical timeline is summarised in Table 2.

**Table 2:** Clinical timeline CRP: C-reactive protein; CT: computed tomography; EBP: epidural blood patch; GONB: greater occipital nerve block; MRI: magnetic resonance imaging; NRS: numerical rating scale; PDPH: post-dural puncture headache; SPGB: sphenopalatine ganglion block

Time	Clinical event
Labour admission (39+6 weeks)	Spontaneous rupture of membranes; request for neuraxial labour analgesia.
Intrapartum	Epidural placement at L3-L4 with 18-G Tuohy needle; technically difficult, three attempts; no recognised dural puncture.
Delivery	Vacuum-assisted vaginal delivery for maternal exhaustion.
Postpartum day 2	Bilateral frontal, postural headache with mild photophobia (NRS 5/10). Clinical diagnosis of PDPH. Conservative treatment commenced (bed rest, oral hydration, caffeine, paracetamol, ketorolac and tramadol).
Postpartum day 4	Fever (38.4°C) and urinary symptoms. Leucocytosis (23.7 × 10⁹/L) and raised CRP (150.6 mg/L). Blood and urine cultures were positive for *Escherichia coli*; pyelonephritis was later confirmed on CT. Intravenous ceftriaxone (1 g) was started once daily.
Postpartum days 5-6	Persistent incapacitating headache (NRS 7/10). Brain MRI with venography normal. EBP deferred because of active bacteraemia.
Postpartum day 6	Bilateral transnasal SPGB (ropivacaine 0.75%, 2 mL per side) followed by bilateral ultrasound-guided GONB (ropivacaine 0.375%, 3 mL per side) after multidisciplinary discussion. NRS decreased from 7/10 to 2/10 within 30 minutes; sustained relief.
Postpartum day 7	Recurrent fever; abdominal CT confirmed acute right pyelonephritis. Subsequent blood cultures were negative; ceftriaxone continued.
Postpartum day 15	Hospital discharge. No headache recurrence.
7 and 30 days after discharge	Telephone follow-up: remained asymptomatic.

## Discussion

This case combines a common therapeutic problem with an uncommon constraint: a typical PDPH in which EBP was deferred because of documented bacteraemia. The 2023 Multisociety International Consensus Guidelines reaffirm EBP as the most effective interventional treatment for moderate-to-severe PDPH but identify active systemic infection as a relative contraindication because of the theoretical risk of meningeal or epidural seeding [4]. Although direct evidence is limited and based on isolated case reports of central nervous system infection following blood patch in patients with bloodstream infection [5], postpartum sepsis with persistent positive cultures and raised inflammatory markers shifts the balance of risk meaningfully compared with that in otherwise well obstetric patients [4].

The choice of peripheral nerve blocks in this setting was pragmatic rather than definitive. SPGB has been described as a minimally invasive treatment for PDPH in obstetric, emergency department and ambulatory settings [7-9], including a Portuguese case series using a similar transnasal technique [9]. Beyond case series, Youssef et al., in a randomised clinical trial in obstetric patients, found both SPGB and GONB to be effective for PDPH after caesarean section [12]. The proposed mechanism of SPGB is modulation of parasympathetic outflow from the pterygopalatine ganglion and reduction in compensatory cerebral vasodilatation, rather than sealing of the dural defect [13]; GONB may complement this through reduction of trigeminocervical input, particularly in headaches with a fronto-occipital distribution [14]. Because these effects are neuromodulatory - interrupting the parasympathetic and trigeminocervical pathways and the self-perpetuating pain cycle rather than providing sustained local anaesthesia - symptom relief may outlast the pharmacological action of a single dose of local anaesthetic [6,13,14]. The 2023 Consensus Guidelines acknowledge that both blocks may be considered when EBP is contraindicated, deferred or declined, while emphasising that the supporting evidence remains weak and based largely on case reports and small case series [4].

The diagnosis of PDPH in this patient was clinical, supported by a classic orthostatic phenotype, fulfilment of ICHD-3 criteria and a rapid response to targeted blocks, after alternative causes had been excluded (see Case Presentation). The blocks were extraspinal and used local anaesthetic alone, eliminating any risk of neuraxial inoculation.

This report has important limitations. No dural puncture was recognised at the time of epidural insertion, so the diagnosis remained clinical and based on phenotype rather than direct observation. Brain MRI showed no imaging signs of intracranial hypotension; however, such signs are insensitive and frequently absent in PDPH, and imaging was performed primarily to exclude alternative pathology rather than to confirm low CSF pressure. The two blocks were performed sequentially in a single session, so the individual contribution of each block cannot be determined; a randomised trial in obstetric PDPH reported that each block was effective when used alone [12]. We used local anaesthetic alone; some authors add a corticosteroid to GONB to prolong its effect, and we cannot exclude that an adjuvant might have further prolonged relief. Pain scores were extracted from clinical records and were not collected with a structured pain protocol. Spontaneous improvement of PDPH around postpartum days 6-7 cannot be entirely excluded, although the temporal relationship with the blocks was striking and the patient had previously failed conservative therapy. We did not offer EBP after blood cultures became negative because the patient was already asymptomatic and clinically improving; whether an earlier offer of EBP after clearance of bacteraemia would have provided additional benefit is uncertain.

From a human-factors perspective, several elements supported safer decision-making in this clinically uncertain situation: a structured multidisciplinary discussion, formal recognition of bacteraemia as a contraindication to EBP, and deliberate re-assessment of the headache before any neuraxial intervention. This case should therefore not be interpreted as evidence that peripheral nerve blocks replace EBP in routine practice; rather, it supports their role as a bridge or rescue strategy when EBP is contraindicated, deferred or declined.

## Conclusions

In this postpartum patient with a clinical diagnosis of PDPH - established by a typical orthostatic phenotype and the exclusion of alternative causes - and concurrent *E. coli *bacteraemia, bilateral transnasal SPGB and bilateral ultrasound-guided GONB provided rapid and sustained symptom relief while avoiding neuraxial intervention during active bloodstream infection. This case should not be interpreted as evidence that peripheral nerve blocks replace EBP in routine practice; rather, it supports their role as a minimally invasive bridge or rescue strategy when EBP is contraindicated, deferred or declined. Prospective studies are needed to define optimal patient selection, the relative contribution of each block and the durability of symptom relief. Until such data are available, careful patient selection and structured multidisciplinary discussion remain essential.
